# Effects of Graphene Nanoplatelets and Cellular Structure on the Thermal Conductivity of Polysulfone Nanocomposite Foams

**DOI:** 10.3390/polym12010025

**Published:** 2019-12-20

**Authors:** Hooman Abbasi, Marcelo Antunes, José Ignacio Velasco

**Affiliations:** Department of Materials Science and Engineering, Technical University of Catalonia (UPC BarcelonaTech), ESEIAAT, C/Colom 11, E-08222 Terrassa, Spain; hooman.abbasi@upc.edu (H.A.); marcelo.antunes@upc.edu (M.A.)

**Keywords:** polysulfone foams, graphene, thermal conductivity, tortuosity, water vapor induced phase separation, scCO_2_

## Abstract

Polysulfone (PSU) foams containing 0–10 wt% graphene nanoplatelets (GnP) were prepared using two foaming methods. Alongside the analysis of the cellular structure, their thermal conductivity was measured and analyzed. The results showed that the presence of GnP can affect the cellular structure of the foams prepared by both water vapor induced phase separation (WVIPS) and supercritical CO_2_ (scCO_2_) dissolution; however, the impact is greater in the case of foams prepared by WVIPS. In terms of thermal conductivity, the analysis showed an increasing trend by incrementing the amount of GnP and increasing relative density, with the tortuosity of the cellular structure, dependent on the used foaming method, relative density, and amount of GnP, playing a key role in the final value of thermal conductivity. The combination of all these factors showed the possibility of preparing PSU-GnP foams with enhanced thermal conductivity at lower GnP amount by carefully controlling the cellular structure and relative density, opening up their use in lightweight heat dissipators.

## 1. Introduction

Polysulfone (PSU) is a high performance thermoplastic with high thermal and chemical stability, excellent strength and toughness, good environmental stress-crack resistance, and inherent fire resistance [1,2]. Additionally, it is resistant to gamma and e-beam radiation due to its high aromatic content [3]. PSU foaming methods such as carbon dioxide dissolution foaming [4,5,6,7,8], extrusion of microcellular polysulfone using chemical blowing agents [9] and PSU membrane formation [10,11,12,13] have been vastly studied. Nevertheless, the addition of thermally conductive nanofillers such as graphene is still incipient [14].

The high aspect ratio of carbon-based nanofillers allows the preparation of polymer-based nanocomposites with high performance and multifunctionality [15]. Their addition to polymers can provide thermal and electrical conductivity at low nanofiller content, overcoming one of the major technological barriers of polymers and enabling their use in applications such as heat sinks [16] and electronic packaging [17]. Additionally, these nanocomposites are suitable candidates as alternatives to conductive polymers that lack thermal stability and proper mechanical performance [18].

The thermal conductivity of nanocomposites is highly dependent on the filler and polymer type [19]. Other factors such as purity and dimension of the filler, polymer crystallinity and measuring methods, explain the scattered data reported for the thermal conductivity of nanocomposites [18,20]. Previous studies of such materials are focused mainly on various types of carbon-based fillers, owing to their intrinsically high thermal conductivity [21,22,23]. Among them, graphene nanoplatelets (GnP) have been some of the most considered nanofillers in recent studies for enhancing the thermal conductivity of polymer-based nanocomposites [24,25,26]. However, results suggest certain constraints regarding the improvement of thermal conductivity, as nanocomposites prepared with GnP have shown limited thermal conductivity even at high filler content [27].

Foaming of these nanocomposites has been the center of attention with the goal of creating novel foams with improved specific properties. In this sense, we have previously investigated thoroughly the preparation and properties of foams based on other high performance thermoplastics like polyetherimide (PEI) reinforced with GnP and carbon nanotubes [28,29,30,31]. The results presented in these works indicate that foaming provided further enhancement of the electrical conductivity by promoting a better dispersion of the nanofillers through the continuous phase of the nanocomposite foams [29].

Likewise, a great interest has appeared very recently regarding the possibility of tuning and enhancing the thermal conductivity of polymer-based foams and hence extend their applicability by generating a more effective thermal conduction network throughout the polymer cell walls by means of guaranteeing a proper distribution and dispersion of thermally conductive nanoparticles, such as GnP. Foaming could hence provide a viable strategy for developing tailored structures to enhance the heat dissipation efficiency of novel lightweight devices. In this sense, PSU nanocomposite foams containing variable concentrations of GnP (up until 10 wt%) were prepared using two foaming methods: water vapor induced phase separation (WVIPS) and scCO_2_ dissolution. The resulting foams were characterized regarding their cellular structure and thermal conductivity. 

The novelties of the present research are (1) the comparison between two very different foaming methods as is the common method of scCO_2_ dissolution foaming with the less common method of water vapor induced phase separation, usually considered for preparing polymeric membranes [32], with obvious advantages of the second one such as the non-requirement of having to melt-compound the material or the possibility of controlling in an easy way the density and cellular structure of the resulting foams; and (2) the consideration for the first time of how the addition of variable amounts of GnP and the developed cellular structure affect the thermal conductivity values through a tortuosity factor that takes into account the complexity of the cellular structure. By considering this tortuosity factor and the relative density as fundamental parameters in the final thermal conductivity, our work shows the possibility of optimizing the thermal conductivity of PSU-GnP foams through the control of their density and cellular structure, ultimately related to the possibility of obtaining conductive foams at lower GnP amounts, for instance for lightweight heat dissipation components.

## 2. Materials and Methods

### 2.1. Materials

PSU pellets (UDEL P-1700) were purchased from Solvay (Brussels, Belgium) with a density of 1.24 g/cm^3^ and a glass transition temperature of 185 °C. The GnP used in this study was acquired from XG Science Inc. (Lansing, MI, USA) with the commercial name of xGnP-Grade M15. These nanoplatelets have a reported thickness of 6–8 nm, an average platelet diameter of 15 µm, a surface area of 120–150 m^2^/g, a density of 2.2 g/cm^3^, and a thermal conductivity of 3000 and 6 W·m^−1^·K^−1^ parallel and perpendicular to the surface, respectively. *N*-methyl pyrrolidone (NMP) used in this study was obtained from Panreac Química SA (Barcelona, Spain) with 99% purity and a boiling and flash points of 202 and 95 °C, respectively.

### 2.2. Foaming Methods

**Figure 1:**
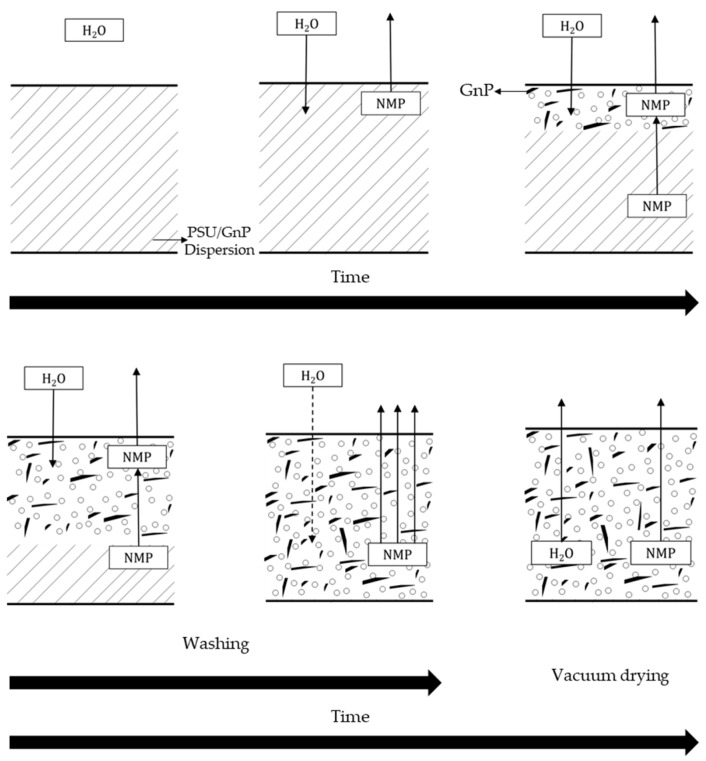
Scheme of the water vapor induced phase separation (WVIPS) process used to prepare PSU-GnP foams from series 1 (15 wt% PSU) and series 2 (25 wt% PSU).

Three series of foams were prepared in this study, the first two using the WVIPS method. In this method, graphene nanoplatelets were initially dispersed in NMP for 30 minutes using a FB-705 ultrasonic processor (Fisher Scientific, Hampton, NH, USA) at maximum amplitude using a 12 mm solid tip probe and 20 kHz, applying a total amount of energy of 90 kJ at 95–130 W. The temperature of this NMP-GnP solution was maintained at 50 °C using an ice-bath. PSU was then dissolved in the NMP-GnP solution at 15 wt% PSU concentration for the first foam series (series 1) and 25 wt% PSU for the second one (series 2) at 50 °C and kept stirring at 450 rpm for 24 h. Foamed samples were respectively coded as “15 PSU x GnP” and “25 PSU x GnP”, with x representing GnP’s weight percentage. In the following step, the solutions were poured on a flat glass and kept at room temperature exposed to air with an average humidity of 75% for 6 days. Foams with variable GnP content (0, 1, 2, 5 and 10 wt%) were prepared for both series. The phase inversion between the solution and water led to the formation of the cellular structure. The resulting foams were then washed in water and ethanol in order to remove the residual solvent and afterwards dried in a vacuum dryer. The process is shown in Figure 1.

A third series of PSU-GnP nanocomposite foams (series 3) were prepared by initially melt-compounding PSU pellets and GnP powder using a Brabender Plastic-Corder (Brabender GmbH and Co., Duisburg, Germany) and foamed using scCO_2_ dissolution foaming inside a high pressure vessel. In order to prepare foams with variable GnP content (0, 0.4, 0.7, 1.0, 1.5 and 2 wt%), a masterbatch containing 50 wt% of ultrasonicated GnP in PSU was initially prepared in an NMP solution and then washed and dried. This masterbatch was then melt-mixed with PSU pellets in the Brabender in order to obtain the desired compositions. Subsequently, the nanocomposites were removed from the Brabender mixing chamber and molded into circular-shaped disks (foam precursors) using a hot-plate press (PL15, IQAP LAP, IQAP Masterbatch Group S.L., Barcelona, Spain) at 250 °C and 80 bar for 14 min. The resulting disks had a nominal thickness of 3 mm and a diameter of 74 mm.

**Figure 2:**
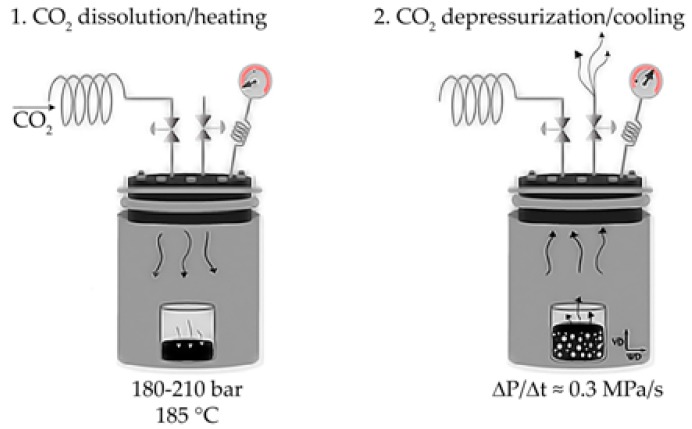
Scheme of the scCO_2_ dissolution foaming process used to prepare PSU-GnP foams from series 3.

Foaming took place in the high-pressure vessel (CH-8610 Uster/Schweiz, Büchiglasuster, Switzerland) by dissolving scCO_2_ at 185 °C and 180–210 bar for 5 h, followed by a sudden depressurization at ~0.3 MPa/s and controlled cooling of the vessel using circulating water. Both steps of scCO_2_ dissolution/heating and depressurization/cooling are shown in Figure 2. Foams from this series were coded as “PSU x GnP” (x representing the weight percentage of GnP). 

### 2.3. Testing Procedure

The foam’s density values were measured using the ISO-845 standard procedure. The cellular structure of the foams was analyzed using a JEOL JSM-5610 (Tokyo, Japan) scanning electron microscope (SEM) applying a voltage of 10 kV and a working distance of 40 mm. Samples were brittle-fractured using liquid nitrogen and later coated with a thin layer of gold by sputter deposition using a BAL-TEC SCD005 (Los Angeles, CA, USA) sputter coater under an argon atmosphere. The values of the average cell size (*Φ*) were measured using the intercept counting method, explained in detail in [33]. Five ×300 magnification SEM micrographs were used for each foam. Cell nucleation density (*N*_0_) and cell density (*N*_f_), which respectively represent the number of cells per volume of unfoamed material and the number of cells per volume of foamed material, were calculated assuming an isotropic distribution of spherical cells according to:

(1) N0=(nA)32(ρsρ),

(2) $$N_{f}=\frac{6}{\pi \Phi^{2}}\left(1-\frac{\rho }{\rho_{s}}\right),$$

where *n* is the number of cells in the micrograph, *A* is its area in cm^2^, and *ρ*_s_ and *ρ* are the solid and foam densities, respectively. 

The thermal conductivity of PSU and PSU-GnP nanocomposites and foams was measured using a C-Therm TCi Thermal Conductivity Analyzer, which employs the Modified Transient Plane Source technique, with a sensor radius of 3.189 mm, optimizing both the power output and measuring time according to the thermal characteristics of each sample (0.005–0.015 W and 15–80 s, respectively). The samples were prepared with the following dimensions: 20 mm × 20 mm × 2 mm (thickness), cutting directly from the obtained foams and flattening the surface using sandpaper.

## 3. Results

### 3.1. Cellular Structure of the Foams

The composition of PSU-GnP nanocomposite foams prepared by WVIPS (series 1 and series 2) and scCO_2_ dissolution (series 3), their respective relative density (*ρ*/*ρ*_s_), and main cellular structure characteristics are presented in Table 1.

**Figure 3:**
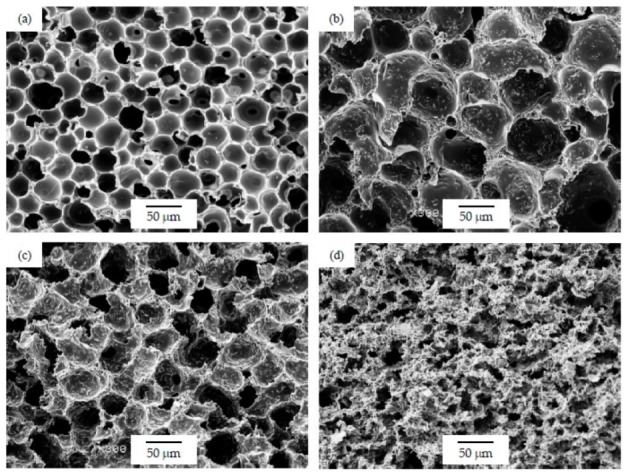
Micrographs at ×300 magnification illustrating the cellular structure of PSU-GnP foams from series 1: (**a**) 15 PSU; (**b**) 15 PSU 2 GnP; (**c**) 15 PSU 5 GnP; and (**d**) 15 PSU 10 GnP.

**Figure 4:**
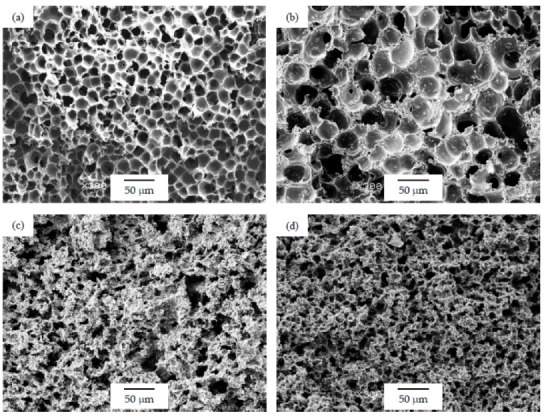
Micrographs at ×300 magnification illustrating the cellular structure of PSU-GnP foams from series 2: (**a**) 25 PSU; (**b**) 25 PSU 2 GnP; (**c**) 25 PSU 5 GnP; and (**d**) 25 PSU 10 GnP.

As can be seen in Table 1, the addition of GnP resulted in a general increase in the average cell size for foams prepared using the WVIPS method when compared to the unfilled PSU foams. The addition of GnP in the first two foam series, that is, those prepared by WVIPS, seemed to affect the kinetics of cell formation due to GnP’s affinity for NMP, which slowed down the process by hindering the phase exchange with water. The greater amounts of GnP in these foams (10 wt% GnP in series 1 and 5 and 10 wt% GnP in series 2) resulted in the formation of foams with open interconnected pores, which could be the result of the mentioned GnP affinity for NMP (see micrographs presented in Figure 3 and Figure 4).

**Figure 5:**
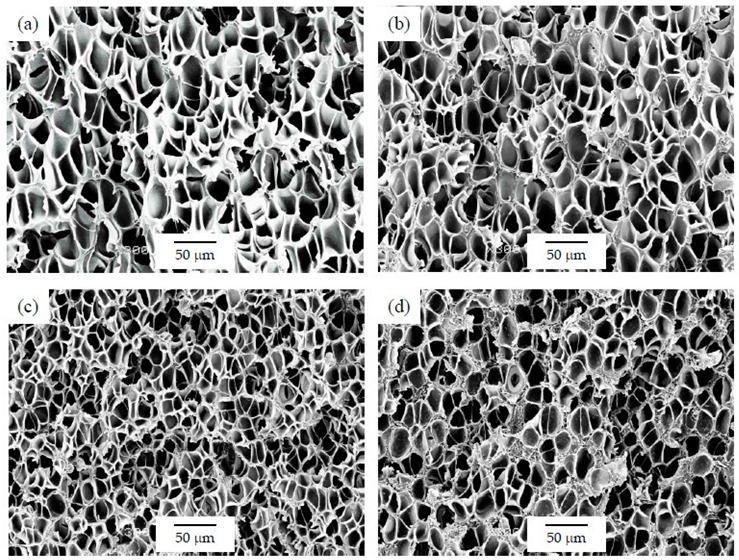
Micrographs at ×300 magnification illustrating the cellular structure of PSU-GnP foams from series 3: (**a**) PSU; (**b**) PSU 0.4 GnP; (**c**) PSU 1 GnP; and (**d**) PSU 2 GnP.

Regarding the cellular structure of foams prepared via scCO_2_ dissolution (series 3), which displayed slightly smaller cell sizes than foams from series 1 and series 2, the addition of GnP resulted in a slight decrease of the average cell size when compared to the unfilled PSU foam (see Figure 5), which could be related to a barrier effect of the platelet-like GnP to the diffusion of scCO_2_. No cell size reduction was observed with further increasing the amount of GnP.

Cell density and cell nucleation density decreased for the first two series of foams with the addition of GnP when compared to the respective unfilled PSU foams. However, by increasing the amount of GnP, these values did not follow a generic pattern. In a similar way, foams prepared by scCO_2_ dissolution displayed a general increase in cell density and cell nucleation density with adding GnP. Nevertheless, no clear trend was observed within these samples related to the increment in the amount of GnP.

### 3.2. Thermal Conductivity

The experimental thermal conductivity (*λ*_exp_) of all foams and their relative density values are presented in Table 2.

**Figure 6:**
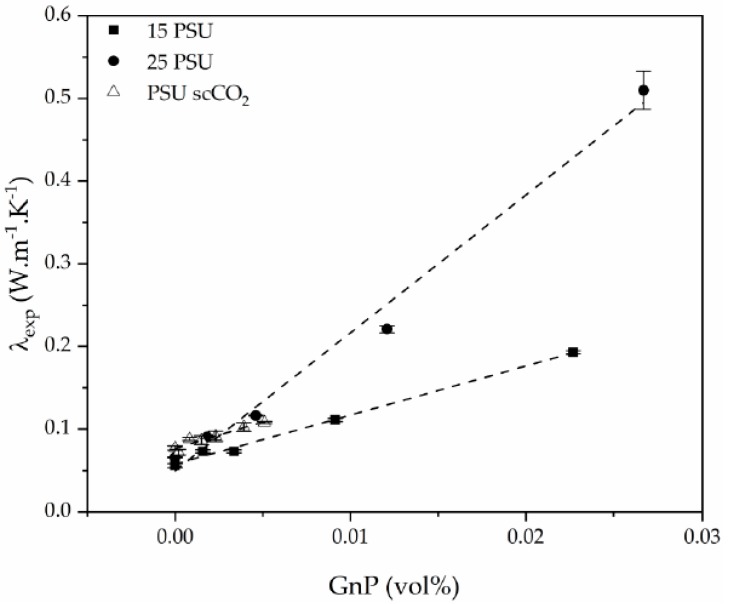
Experimental thermal conductivity enhancement of PSU and PSU-GnP nanocomposite foams with increasing GnP amount.

**Figure 7:**
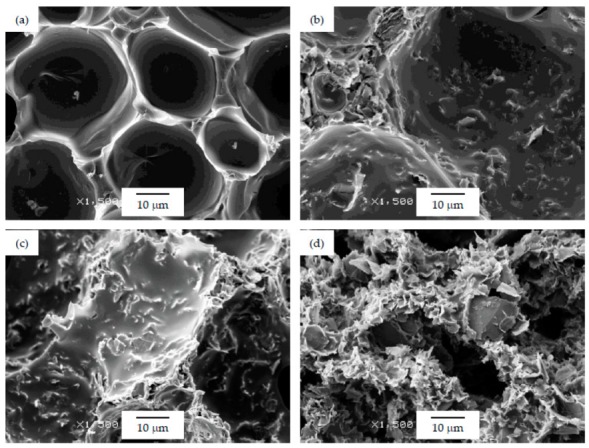
Micrographs at ×1500 magnification illustrating the cell walls of PSU-GnP foams from series 1: (**a**) 15 PSU; (**b**) 15 PSU 2 GnP; (**c**) 15 PSU 5 GnP; and (**d**) 15 PSU 10 GnP.

**Figure 8:**
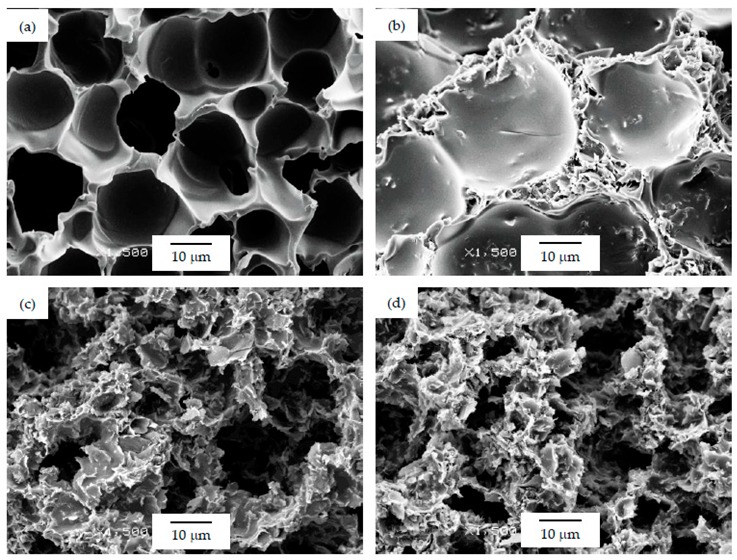
Micrographs at ×1500 magnification illustrating the cell walls of PSU-GnP foams from series 2: (**a**) 25 PSU; (**b**) 25 PSU 2 GnP; (**c**) 25 PSU 5 GnP; and (**d**) 25 PSU 10 GnP.

**Figure 9:**
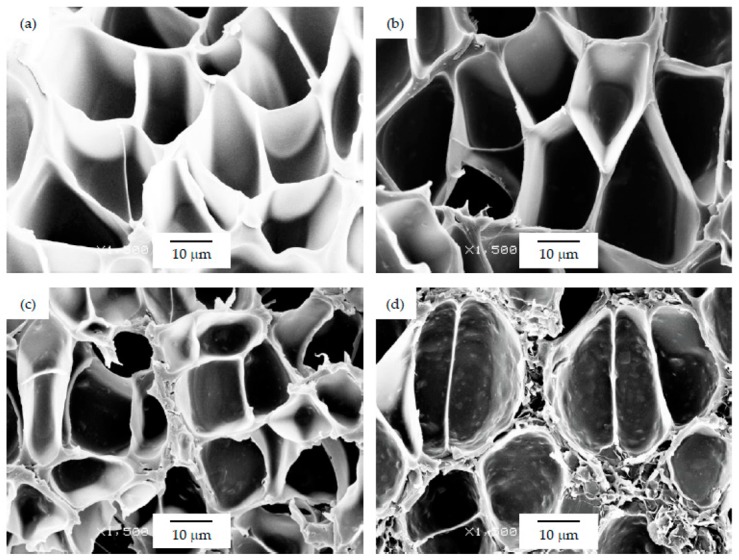
Micrographs at ×1500 magnification illustrating the cell walls of PSU-GnP foams from series 3: (**a**) PSU; (**b**) PSU 0.4 GnP; (**c**) PSU 1 GnP; and (**d**) PSU 2 GnP.

**Figure 10:**
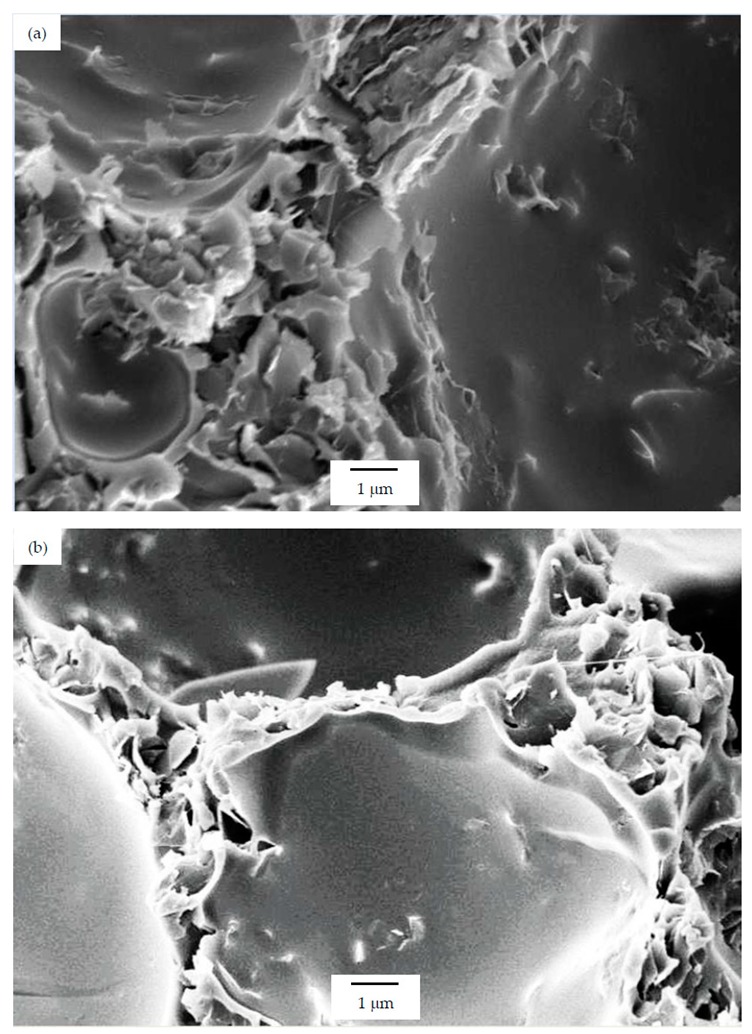

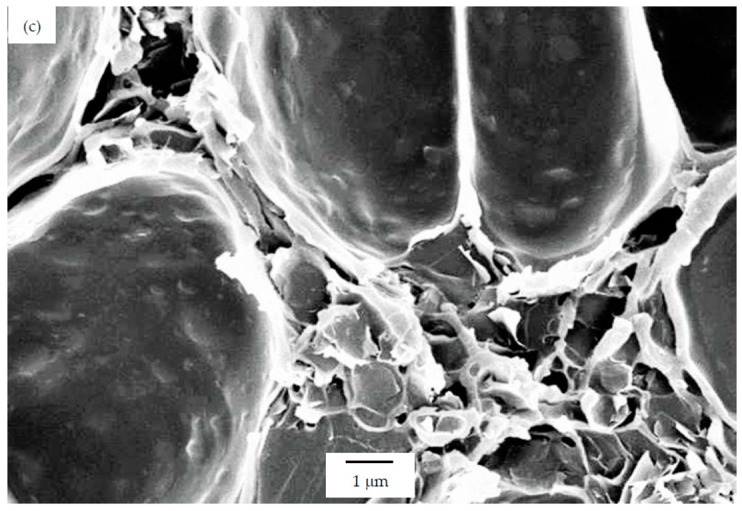
High-magnification micrographs (×5000) illustrating the distribution and dispersion of GnP throughout the cell walls of PSU-GnP foams with 2 wt% GnP: (**a**) 15 PSU 2 GnP; (**b**) 25 PSU 2 GnP; and (**c**) PSU 2 GnP.

As can be seen, there was a direct relation between the thermal conductivity and the GnP amount, as all PSU-GnP nanocomposite foams displayed an increasingly higher thermal conductivity with incrementing the amount of GnP (see Figure 6), owing to the inherently high thermal conductivity of GnP and the higher probability of physical contact between graphene nanoplatelets (see the ×1500 magnification micrographs presented in Figure 7, Figure 8 and Figure 9 and especially the comparative high magnification micrographs presented in Figure 10 showing the distribution and dispersion of GnP throughout the cell walls of PSU-GnP foams). No significant differences were observed in terms of GnP dispersion between foams with 2 wt% GnP prepared by the two foaming methods (Figure 10). The experimental thermal conductivity of each foam series followed a linear trend with the volume percentage of GnP (R^2^ ≥ 0.93) with different slopes for each of the series (Figure 6). 

**Figure 11:**
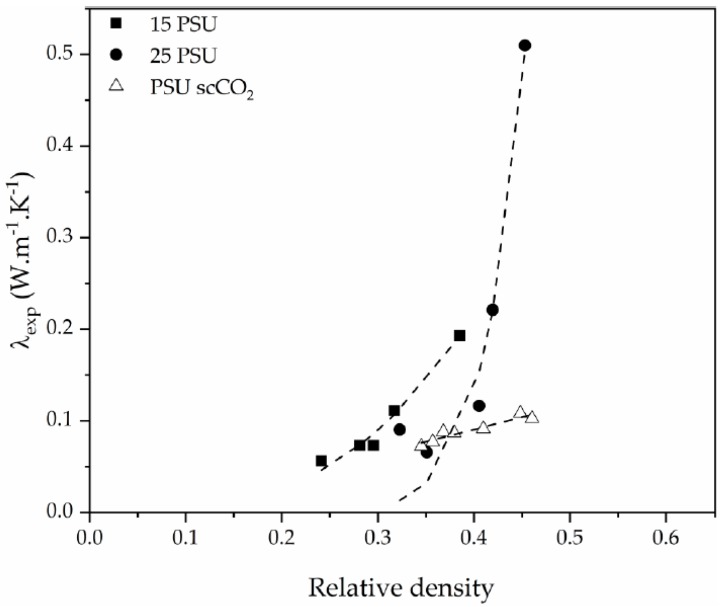
Experimental thermal conductivity enhancement of PSU and PSU-GnP nanocomposite foams with increasing relative density.

Relative density also played a significant role, as thermal conductivity augmented with increasing relative density (Figure 11), especially in the case of PSU-GnP foams from series 2. This increment was related to the increasingly higher importance of thermal conduction through the solid phase, formed by a combination of PSU and thermally conductive GnP.

**Figure 12:**
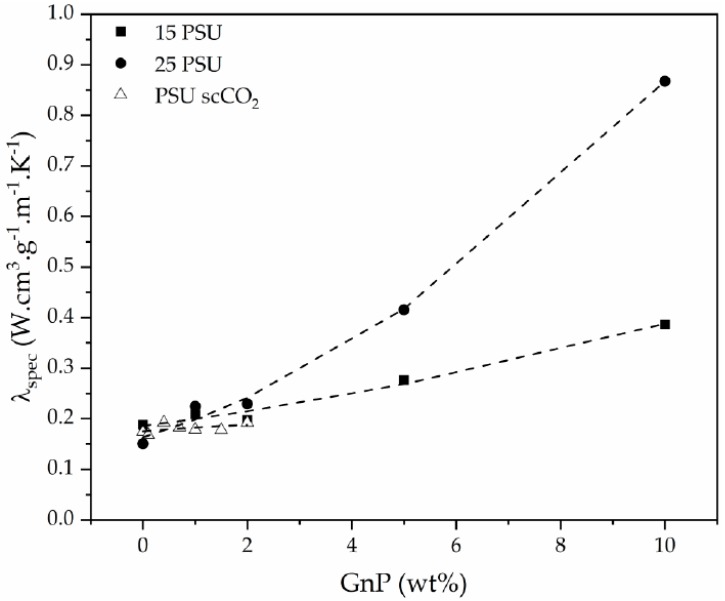
Specific experimental thermal conductivity evolution of PSU and PSU-GnP nanocomposite foams with increasing GnP amount.

The effect of GnP influence on the thermal conductivity of the foams regardless of the changes in relative density can be observed in Figure 12 by representing the specific experimental thermal conductivity (*λ*_spec_), defined as the quotient between the experimental thermal conductivity and the density of the foam, as a function of GnP amount.

The thermal conductivity of nanocomposite foams (*λ*_f_) can be assumed as a contribution of four factors [34]: thermal conduction through the solid nanocomposite (*λ*_s_), conduction through the gas phase (*λ*_g_), convection through the cells (*λ*_cv_) and radiation through the cell struts and across the cell voids (*λ*_r_):

(3) $$\lambda_{f}=\lambda_{s}+\lambda_{g}+\lambda_{\mathrm{cv}}+\lambda_{r}.$$

The thermal conductivity due to convection (*λ*_cv_) is only considered significant when the Grashof number is greater than 1000, which requires a minimum cell size of 10 mm [35,36]; therefore, it can be ignored for these foams.

Regarding the contribution of radiation (*λ*_r_), the heat flux going through the foam in radiation form can be modeled as radiation across a series of parallel opaque surfaces with a separation equal to the average cell size (*Φ*) according to [34]:

(4) $$\lambda_{r}=4\frac{\varepsilon }{2-\varepsilon }\sigma T^{3}\Phi ,$$

where *ε* is the cell wall emissivity and *σ* is the Stefan–Boltzmann constant. The radiation contribution can also be disregarded for the foams presented in this work, as all of them are black due to the presence of GnP and have been prepared with relatively high thicknesses. Since the conductive GnP particles play a key role in the final thermal conduction behavior of the foams, a two-phase model was suggested for predicting thermal conductivity:

(5) $$\lambda_{f}=\lambda_{g}V_{g}+\xi \left(\lambda_{c}V_{c}\right),$$

in which *λ*_f_ represents the thermal conductivity of the foam, *λ*_g_ corresponds to the conductivity of the gas in the cells (*λ*_air_ = 0.026 W·m^−1^·K^−1^ [37]), *λ*_c_ represents the conductivity of the solid nanocomposite phase (PSU-GnP, experimentally determined to be between 0.210 W·m^−1^·K^−1^ for unfilled PSU and 0.282 W·m^−1^·K^−1^ for PSU-GnP foams containing the highest amount of GnP) and *ξ* is a parameter related to the tortuosity, which depends on the complexity of the foam’s structure. *V*_g_ and *V*_c_ correspond to the volume fraction of the gas and solid phase, respectively. 

As can be seen when comparing the SEM micrographs presented in Figure 3, Figure 4 and Figure 5, and in Figure 7, Figure 8 and Figure 9, and the cellular structure characteristics presented in Table 1, the changes in cellular structure could alter the tortuosity factor *ξ*, directly affecting the values of thermal conductivity; therefore, the *ξλ*_c_ product was calculated from the experimental thermal conductivity values for each foam using the following equation and presented in Table 3:

(6) $$\xi \lambda_{c}=\frac{\lambda_{f}-\lambda_{g}V_{g}}{V_{c}}.$$

The effect of changes in tortuosity could be observed in foams with similar composition. In the case of foams prepared using the WVIPS method, the ones with an open-cell structure (15 PSU 10 GnP, 25 PSU 5 GnP and 25 PSU 10 GnP) showed considerably lower tortuosity (higher *ξλ*_c_ values) and, as a consequence, displayed higher values of thermal conductivity. For foams with closed-cell structure, the value of the specific thermal conductivity decreased with decreasing cell size and hence increasing cell nucleation density, directly related to a higher tortuosity.

The drawback in the two-phase model presented in Equation (5) is that the effect of foam’s density on the thermal conductivity of the foam is not clearly introduced. The already shown increase in thermal conductivity of the foam by incrementing relative density (Figure 11) can be explained by a power law expression similar to the one suggested by Gibson and Ashby for predicting the mechanical performance of cellular solids [35]:

(7) $$\frac{\lambda_{f}}{\lambda_{c}}=K{\left(\frac{\rho }{\rho_{s}}\right)}^{n}.$$

In this equation, if we assume *K* as being a variable equivalent to the tortuosity parameter *ξ*, exponent *n* can be seen as representing the foaming efficiency in the final thermal conductivity, being related to the relative density according to:

(8) $$n=\frac{\left[\ln\left(\lambda_{f}\right)-\ln\left(\xi \lambda_{c}\right)\right]}{\left[\ln\left(\frac{\rho }{\rho_{s}}\right)\right]}.$$

The values of *n* have been calculated for each foam and are presented in the final column of Table 3. As the values of *n* suggest, the highest efficiency corresponds to the foams from series 2, i.e., foams prepared by the WVIPS method with a 25% of PSU.

**Figure 13:**
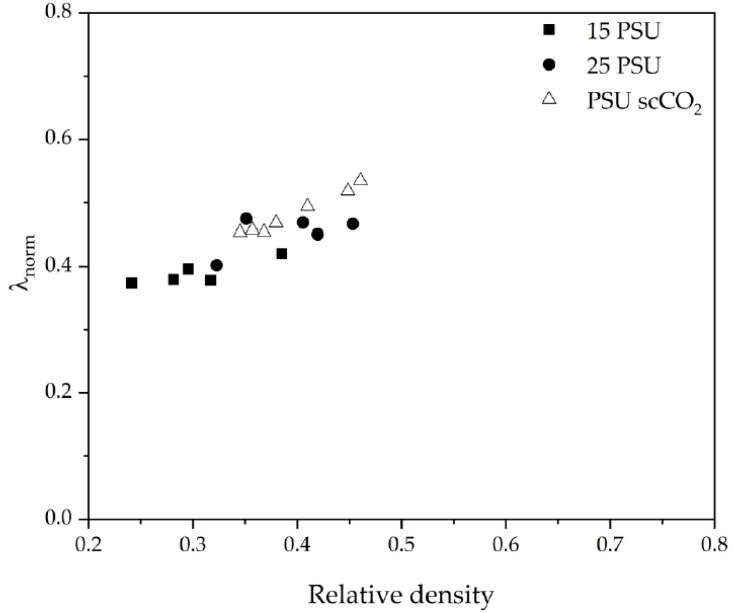
Normalized thermal conductivity evolution with relative density for PSU and PSU-GnP nanocomposite foams.

These results, combined with the previous analysis on the tortuosity and cellular structure of the foams, could lead to an optimization of the thermal conductivity of PSU-GnP nanocomposite foams by controlling their density and cellular structure, and hence to the achievement of highly conductive foams at lower amounts of GnP. This can be more clearly seen in Figure 13 by representing the normalized thermal conductivity of the foams (*λ*_norm_), calculated by dividing *λ*_f_ by (*ξλ*_c_), as a function of relative density.

## 4. Conclusions

Three series of PSU-GnP nanocomposite foams with variable amounts of GnP (between 0 and 10 wt%) were prepared using two foaming methods: WVIPS and scCO_2_ dissolution. Foams prepared by the WVIPS method presented relative densities between 0.24–0.39 for series 1 (foams prepared with 15% PSU) and between 0.35–0.45 for series 2 (foams prepared with 25% PSU), while foams prepared by scCO_2_ dissolution (series 3) displayed relative densities between 0.36–0.45. In all series, relative density increased with incrementing the amount of GnP. 

In terms of cellular structure, foams from series 1 and series 2 showed higher average cell sizes and hence lower cell densities and cell nucleation densities with the addition of GnP. Among these foams, those with lower amounts of GnP displayed a homogenous closed-cell structure, whereas those with a higher GnP concentration (10 wt% GnP in series 1 and 5 and 10 wt% GnP in series 2) showed an open-cell interconnected structure. These results suggest that the presence of GnP affected the kinetics during the phase separation exchange process by slowing it down due to the affinity of GnP for NMP. In the case of foams prepared via scCO_2_ dissolution, an opposite trend was observed with only slight changes. The average cell size of foams from series 3 slightly decreased by adding GnP, which could be the result of a physical barrier effect of the platelet-like GnP to the diffusion of CO_2_ in PSU.

The thermal conductivity of the foams was affected mainly by the presence of GnP, the cellular structure, and the density of the foams. The thermal conductivity showed a linear increasing trend with increasing GnP volume fraction, as expected due to the intrinsically high thermal conductivity of GnP. Additionally, the tortuosity of the cellular structure, also directly related to the added amount of GnP, influenced the final value of thermal conductivity by affecting the path of conduction. The highest values of thermal conductivity corresponded to foams with an open-cell interconnected structure. Moreover, density played a key role, as thermal conductivity followed a power law relation with relative density. The combination of these factors showed that PSU-GnP foams can be prepared with higher thermal conductivity at a lower amount of GnP by carefully controlling their cellular structure and relative density. 

## Figures and Tables

**Table 1:** Composition, relative densities and cellular structure characteristics of PSU and PSU-GnP nanocomposite foams.

Foam Series	Foam Code	Density (g/cm^3^)	Relative Density	*V*_g_	*V*_PSU_	*V*_GnP_	*Φ* (μm)	*N*_0_ (cells/cm^3^)	*N*_f_ (cells/cm^3^)
Series 1	15 PSU	0.299	0.24	0.759	0.241	0.000	29.7	1.9 × 10^8^	5.5 × 10^7^
	15 PSU 1 GnP	0.351	0.28	0.719	0.280	0.002	55.5	3.3 × 10^7^	8.1 × 10^6^
	15 PSU 2 GnP	0.370	0.30	0.705	0.292	0.003	56.7	3.2 × 10^7^	7.5 × 10^6^
	15 PSU 5 GnP	0.402	0.32	0.683	0.308	0.009	50.1	5.1 × 10^7^	1.0 × 10^7^
	15 PSU 10 GnP	0.500	0.39	0.615	0.362	0.023	Open cell	-	-
Series 2	25 PSU	0.435	0.35	0.649	0.351	0.000	19.9	5.2 × 10^8^	1.5 × 10^8^
	25 PSU 1 GnP	0.402	0.32	0.677	0.321	0.002	21.2	3.7 × 10^8^	1.3 ×10^8^
	25 PSU 2 GnP	0.507	0.41	0.595	0.401	0.005	33.9	1.0 × 10^8^	2.9 × 10^7^
	25 PSU 5 GnP	0.532	0.42	0.580	0.408	0.012	Open cell	-	-
	25 PSU 10 GnP	0.588	0.45	0.547	0.426	0.027	Open cell	-	-
Series 3	PSU	0.443	0.36	0.643	0.357	0.000	19.1	2.1 × 10^8^	1.8 × 10^8^
	PSU 0.1 GnP	0.428	0.35	0.655	0.345	0.000	17.3	2.5 × 10^8^	2.4 × 10^8^
	PSU 0.4 GnP	0.457	0.37	0.632	0.367	0.001	13.9	3.4 × 10^8^	4.5 × 10^8^
	PSU 0.7 GnP	0.472	0.38	0.620	0.378	0.002	15.1	2.7 × 10^8^	3.5 × 10^8^
	PSU 1 GnP	0.510	0.41	0.590	0.407	0.002	13.1	4.1 × 10^8^	5.1 × 10^8^
	PSU 1.5 GnP	0.575	0.46	0.539	0.457	0.004	13.8	3.7 × 10^8^	4.0 × 10^8^
	PSU 2 GnP	0.561	0.45	0.552	0.443	0.005	14.9	2.1 × 10^8^	3.1 × 10^8^

**Table 2:** Experimental thermal conductivity values of PSU and PSU-GnP nanocomposite foams.

Foam Series	Foam Code	Relative Density	*λ*_exp_ (W·m^−1^·K^−1^)
Series 1	15 PSU	0.24	0.056
	15 PSU 1 GnP	0.28	0.073
	15 PSU 2 GnP	0.30	0.073
	15 PSU 5 GnP	0.32	0.111
	15 PSU 10 GnP	0.39	0.193
Series 2	25 PSU	0.35	0.066
	25 PSU 1 GnP	0.32	0.090
	25 PSU 2 GnP	0.41	0.116
	25 PSU 5 GnP	0.42	0.221
	25 PSU 10 GnP	0.45	0.510
Series 3	PSU	0.36	0.077
	PSU 0.1 GnP	0.35	0.072
	PSU 0.4 GnP	0.37	0.088
	PSU 0.7 GnP	0.38	0.087
	PSU 1 GnP	0.41	0.091
	PSU 1.5 GnP	0.46	0.102
	PSU 2 GnP	0.45	0.108

**Table 3:** Tortuosity effect (*ξλ*_c_) and foaming efficiency (*n*) influence on the thermal conductivity of PSU and PSU-GnP nanocomposite foams.

Foam Series	GnP (wt%)	*ξλ*_c_ (W·m^−1^·K^−1^)	Relative Density	*n*
Series 1	0	0.150	0.24	0.692
	1	0.192	0.28	0.764
	2	0.184	0.30	0.760
	5	0.294	0.32	0.847
	10	0.459	0.39	0.909
Series 2	0	0.138	0.35	0.712
	1	0.225	0.32	0.806
	2	0.248	0.41	0.840
	5	0.490	0.42	0.918
	10	1.093	0.45	0.964
Series 3	0	0.168	0.36	0.760
	0.1	0.159	0.35	0.744
	0.4	0.194	0.37	0.791
	0.7	0.185	0.38	0.785
	1.0	0.185	0.41	0.791
	1.5	0.192	0.46	0.808
	2.0	0.209	0.45	0.821
